# “That kind of information is crucial to get across”: co-developing a sexual assault support website with survivors and support providers

**DOI:** 10.1080/17482631.2025.2592404

**Published:** 2025-11-28

**Authors:** Maria Hardeberg Bach, Sascha Nikoline Strauss Krogh, Maj Hansen

**Affiliations:** a THRIVE, Department of Psychology, University of Southern, Denmark; b Center for Sexual Assault, Aarhus University Hospital, Denmark

**Keywords:** Sexual violence, informal support providers, formal support systems, eHealth, action research

## Abstract

**Purpose:**

Although eHealth can play a vital role in the lives of people affected by sexual violence, research indicates that eHealth applications often do not meet these users’ needs.

**Methods:**

This study used action research to develop a national website in Denmark for sexual assault support. This process involved collaboration between employees at a sexual assault center and university researchers, as well as meetings and interviews with representatives from website user groups (survivors and support providers).

**Results:**

Formal interviews were analyzed using thematic analysis. Results indicate that websites are used to define sexual assault (Theme 1), that information to counter rape myths is essential (Theme 2), that all those affected by sexual violence must be addressed, not just survivors (Theme 3), that wording and labels matter (Theme 4), and that interactive materials are desired (Theme 5). A new sexual assault website was developed, resulting in a more contemporary, accessible, and inclusive website (e.g., by broadening and diversifying descriptions of sexual violence; by addressing the pain and suffering experienced by survivors’ loved ones).

**Discussion:**

This study illustrates that action research in this area is feasible and that survivors and support providers can contribute significantly to the creation of new interventions.

Sexual violence is pervasive worldwide (Dworkin et al., 2021), inflicting profound harm on survivors’ physical and mental health, necessitating specialized interventions (Dworkin et al., 2023). Being a survivor of sexual violence is a highly stigmatized identity in most cultures, and disclosing sexual assault therefore comes at a risk (e.g., receipt of negative reactions, loss of privacy, risk of retaliation) (Kennedy & Prock, 2018). As a result, many survivors chose to cope in isolation and silence (Monroe et al., 2005; Ullman et al., 2020). Among survivors who do come forward at some point, many experience obstacles in relation to obtaining formal support (Zinzow et al., 2021). Not all survivors know where to turn for help, and survivors often have complex, co-occurring needs, which may result in having to navigate multiple sectors at once (e.g., medical, mental health, social, criminal justice sector) (Campbell, 2013; Gagnon et al., 2018). Having located desired services, survivors may still not be able to access them due to, for example, service fees, long waitlists, long distances to services, transportation issues, inability to take time off work, buildings that are non-compliant with disability acts, and lack of multilingual services (Bach et al., 2023).

Over the years, and in an attempt to improve some of these barriers, eHealth interventions have been increasingly used for sexual assault service provision (Bach et al., 2023). Broadly defined, eHealth refers to the provision of healthcare using the internet and hence covers many types of healthcare practices (e.g., patient consultations, prescription of medicine) and technologies (e.g., web and mobile apps) (da Fonseca et al., 2021). eHealth is one of the fastest growing areas in health and has dramatically changed the healthcare field (Oh et al., 2005; WHO, 2019). Research indicates that many survivors view eHealth positively, with some even preferring it to traditional face-to-face services (e.g., Carretta et al., 2016). Reported advantages include a heightened sense of anonymity and increased flexibility, as online services can often be accessed from any location and at any time—features that proved invaluable during COVID−19 lockdowns (Moylan et al., 2021; Zheng & Gray, 2014).

The present research took place at one of the largest centers for sexual assault survivors in Denmark. The Danish Sexual Assault care Centers (SACs) provide forensic medical examination and psychosocial support to survivors approximately 15 and upwards in hospitals throughout the country. The first Danish SAC was established in 1999, based on service models including the well-known Sexual Assault Response Team (SART) model (Bramsen et al., 2009). Although some variation between individual SACs exists, psychosocial support typically involves access to anonymous, 24/7 helplines, short- to medium length psychological treatment provided by psychologists, and accommodation of practical needs from social workers (e.g., facilitation of sick leave). Most survivors are eligible for psychological treatment, but some are referred elsewhere due to co-existing needs or because more suitable treatment exists (e.g., children, survivors with severe addiction or mental health problems). SACs are integrated into the national healthcare system, and like other hospital-services in Denmark, services are free of charge. No police involvement is needed to access SACs. As in most countries, face-to-face consultations were replaced with video consultations during COVID−19, and SAC providers generally had little to no time to prepare for this new mode of service provision. Concurrently, more survivors than ever before were contacting SACs, resulting in waiting times for up to two years for treatment. SAC providers therefore expressed a need to strengthen the digital presence and reach of SACs. To tackle the abovementioned issues, a team of university researchers and SAC employees embarked on a collaborative project to update and expand eHealth use at a Danish SAC. In particular, the present paper focuses on efforts to redesign and re-launch the center’s website www.voldtaegt.dk (“rape.dk” translated to English. Hence forward referred to as the “SAC website” or simply “the website”). Although the website in question is regarded as the main e-resource on sexual assault in the country, it had not been updated regularly for several years, resulting in inconsistencies, inaccuracies, and outdated information.

Prior research in this area is relatively limited but there is evidence to suggest that eHealth applications for sexual assault often do not meet the needs of users. Accessibility issues include the presence of user fees for some applications, apparent absence of disability enhancing features, reliance on text-best communication, and predominance of monolingual applications (Hicks et al., 2017; Sorenson et al., 2014; Wood et al., 2020). Concerns about relevance have also been raised, as not all survivors are comfortable with technology, its use is not accepted in all societies, and many applications appear to prioritize certain survivors and forms of violence above others (e.g., cis women) (Sleath et al., 2025; Sorenson et al., 2014). There has therefore been a call for innovative research in this area, including qualitative studies and user involvement (Bach et al., 2023; Bakker et al., 2020; Sleath et al., 2025). The redesign and re-launch of the website was therefore based on continuous involvement of different website users to better understand their experiences and needs in this area. Although the SAC website is open to anyone (e.g., students, media), it is primarily intended for adult and adolescent survivors, corresponding to the population served at SACs, and secondly informal and formal support providers (alternative web-resources for child sexual abuse exist). Due to global similarities in sexual assault service provision and increased reliance on eHealth everywhere (Walby et al., 2015), we believe the present study is relevant for anyone seeking to develop eHealth, as well as those interested in conducting collaborative research in general.

## Method

### Action research

The present study was inspired by action research, which can be seen as the simultaneous process of taking action and conducting research (Rowell et al., 2015). In particular, we wanted to generate knowledge about website users’ experiences and needs, while also using this knowledge to develop a concrete product: A website. Action research encourages researchers and non-researchers to participate in shared learning and decision-making (Kemmis et al., 2014). To establish this kind of meaningful, authentic partnership, the team was provided an office space at the SAC and had daily contact throughout the project. The project team consisted of two university-based researchers and a SAC employee (the authors), who drew on other professionals including web developers, graphic designers, and so forth. An early step in the research project involved conducting a review of the international literature pertaining to eHealth for sexual assault (Bach et al., 2023) to ground the research in existing research. Subsequently, informal meetings and formal interviews with website users were held throughout the project to identify additional user experiences and needs rooted in the local context. As many user recommendations as possible have been implemented on the new website (within our budget, timeline, and hospital regulations), resulting in a new website in 2024 (same web domain). The website is currently being evaluated by additional website users and will be further amended, reflecting the dynamic and collaborative process, which is at the core of action research. This process can be described as “a spiral of self-reflective cycles” consisting of 1) planning a change, 2) implementing a change, 3) observing and reflecting on the change, and 4) repeating the process (Kemmis et al., 2014).

### Participants

For this part of the research project, eight website users participated in formal interviews. Interview participants were recruited from the SAC. Prior knowledge of the SAC website was not required, and neither was engagement with any type of formal support. Flyers for survivors and informal support were placed in the waiting room and handed out by SAC providers, inviting those interested to share their thoughts and perspectives on the SAC’s online resources, including the original website. Formal support providers were invited by email. Inclusion criteria for survivors and informal support providers were being over 18 and having experienced a sexual assault personally or being a close friend/relative/partner to a survivor of sexual assault. For this part of the research project, two survivors (“Julie” and “Louise” in their 20 s) and two mothers of teenage survivors (“Linda” in her 40 s and “Helen” in her 50 s) signed up. All types of formal support providers working with sexual assault were eligible to participate in the study. Four formal support providers affiliated with the SAC responded to the email invite and participated in the study (two psychologists, one social worker, and one administrative worker who interacted with survivors). All participants identified as female.

### Data collection

A semi-structured interview guide focusing on the experiences and needs of website users was developed for each of the three user groups based on the suggestions in Hennink et al. (2020). In particular, we showed participants sections of the original SAC website and asked about their history of using the SAC website and other websites for sexual assault; what kind of information participants were seeking at the time, how they perceived different sections of the SAC website; how it could be improved; and what other eHealth features, if any, they wanted. While most questions were open and broad, participants were also asked to give very concrete feedback or co-write some sections of the new website.

Due to the sensitivity of the topic, survivors and informal support providers were interviewed individually. Formal support providers knew each other in advance and felt comfortable participating in group interviews (Flick, 2018). Interviews were conducted in Danish in 2023−24 by the first author. Participation was voluntary. All participants signed a consent form before the interview took place. All interviews took place at the SAC, a familiar and safe space for participants. All interviews were audio recorded. Individual interviews were transcribed verbatim. For group interviews, one member of the project group led the interview, while the other took extensive notes. Quotes presented in the manuscript have been translated to English by the first author. All participants were given an alias. The present study was conducted according to the principles of the Declaration of Helsinki (WMA, 2024). All necessary ethical and legal approvals according to Danish law were granted for conducting the present study and publishing results. The study was registered with the Research and Innovation Organization at the University of Southern Denmark (clearance number 11.429), which involves legal assistance to ensure lawful data processing and safeguarding of research participants’ safety and rights. As the study does not involve human biological material, it was exempt from further ethical review by the Danish National Committee on Health Research Ethics.

### Data analysis

Analysis was inspired by reflexive thematic analysis (Braun & Clarke, 2006; Braun et al., 2022). This strategy was selected because it provides a systematic yet flexible approach to data analysis. Our approach was mostly inductive and experiential, since we were oriented towards participants’ points of view, rather than seeking links in the data to predefined theoretical concepts. We also consider our position to be informed by critical realism (Pilgrim, 2014). Analysis was an iterative process but overall unfolded as follows: First, the transcripts and notes were read to re-orient ourselves towards the data (phase 1). Initial coding commenced, which involved tagging and naming sections of the data that were considered meaningful in relation to the research purpose (phase 2). Because the goal of the research project was to use participants’ responses to update the website accordingly, the level of coding was more semantic than latent (i.e., focused on explicit meaning rather than unspoken, “deeper” meanings). Next, we started developing themes (i.e., significant concepts or ideas evident throughout the initial codes) (phase 3). Finally, phase 4 and 5 consisted of reviewing potential themes (e.g., returning to the dataset to determine if themes were still appropriate), before organizing and naming the final themes (Terry et al., 2017). Analysis was conducted by the first author through systematic engagement with the data (transcripts, notes) but further shaped and developed by extensive discussions in the author group.

## Results

Five themes covering various aspects of the participants’ experiences and needs were identified and presented below.

### Theme 1: website function

Although formal support providers had all used the SAC website prior to the interview (e.g., to locate information and statistics about SACs and sexual assault), they mainly viewed the SAC website as a platform to inform about the presence and purpose of SACs to others (survivors, informal providers, external service providers, the public). Survivors and informal support providers had all accessed the SAC website at some point following the assaults and prior to the interview. All four came across the website by searching the internet for terms like “rape” and “sexual assault”. Julie and Louise (survivors) used the website to a) determine whether what they had experienced was sexual assault, b) evaluate if experienced distress was related to assault, and c) discover what SACs offered and whether they qualified for this help. According to Julie: “I read virtually anything I could find about sexual assault (…) I just had to know if I was entitled to get help. If this [assault] was real”. Julie and Louise had both initially doubted the legitimacy of their assault because it took place in a “party context” and because they had not physically resisted:

When I think “sexual assault” it’s that, you know, you’ve been attacked, and you can’t physically overpower them. You’ve done everything you could, kicked and screamed (…) I didn’t scream, I didn’t hit, I didn’t anything (…) I didn’t feel like I deserved help” (Louise).

Louise discussed her experience with close friends and family members, but felt she needed formal validation to determine if it was a sexual assault: “My friends and family told me it was [sexual assault], but I didn’t believe them. So, seeing it [assault definition] in black and white on an official website was immensely important to me”. Apart from naming their experience, survivors used the website to evaluate if experienced distress resulted from the assault: “I primarily used it [the website] to sort of diagnose myself”. Informal support providers (Helen and Linda) also accessed the internet in search of information and support, including advice on what actions needed to be taken following a sexual assault (of a minor). When Helen learned about her daughter’s assault from someone who she had entrusted with her experience, Helen consulted the internet right away: “I searched online because I had to ask someone: What the hell do I do now?”.

### Theme 2: helpful content and features

Participants were overall positive about using the internet, including the SAC website, for information and support for sexual assault. All participants considered website material aimed at validating survivors’ experiences essential. This involved normalizing survivors’ actions and reactions before, during, and after sexual assault. Both survivors shared that they struggled with deep-rooted and long-lasting feelings of shame and self-blame for their assault (e.g., not physically resisting the assault). Survivors’ main message to other survivors was that sexual assault is never the survivor’s fault, regardless of survivors’ actions. Providing information to counter rape myths was seen as helpful in this context (e.g., many survivors do not physically resist the assault). Due to the centrality of shame and blame in societal narratives surrounding sexual assault, participants also considered material on how to best respond to disclosures of sexual assault to be helpful. In this context, Helen and Linda (informal providers) shared that their initial reaction to learning about their daughters’ assaults were shaped by more limited knowledge about sexual assault at the time. According to Helen:

I asked her a hundred things that I later realized I shouldn’t have (…) As a mother, you think: Why didn’t she come to us straight away? And then you ask about the situation: Why didn’t you scream? Did you resist?

Helen had since read about rape myths on the website, which made her see her daughter’s actions in a new light. She therefore concluded: “That kind of information is crucial to get across [on websites]”.

Participants of all kinds liked that the website was structured into different sections for different users (i.e., survivors, informal support providers, professionals outside SACs, the general public). Nevertheless, several participants warned against categorizing certain types of information as being strictly for professionals, because doing so implied that other groups could not “comprehend” its contents. Participants identified various advice on the website as helpful—for instance, recommendations that survivors seek professional support. However, some expressed concern that such advice might unintentionally create pressure or feelings of inadequacy if not carefully worded. Specifically, it could imply that “right” or “wrong” ways to process and respond to an assault exist. As explained by Louise, for example: “Some of these [advice on website] can seem like a checklist: You have to do it this way to do it right. But (…) what helps is individual”.

### Theme 3: unhelpful content and features

Participants of all kinds agreed that the original SAC website was difficult to navigate and information heavy (e.g., text too small, too many sections, too many clicks, too much text, identical content across sections for different user groups). Participants generally emphasized the importance of clear and short communication as many survivors are in a state of shock and emotional exhaustion: “You shouldn’t use too much text [on website]. Your brain is pretty stressed out already [because of assault]” (Julie, survivor). Some participants also worried about people with low reading abilities in this context. Audio/visual material was seen as more accessible and comforting: “Having a narrator makes you feel safer (…) Going into this you feel so alone in the world. So having a human being telling you about something, then you’re already kind of two” (Helen, mother).

Informal and formal support providers noted that there was limited, if any, information on the website about informal support providers’ own feelings and reactions to the assault of a loved one. Both informal support providers described their experience as extremely painful. According to Helene: “It’s been a year [since assault] and I’ve held my breath for a year” and Linda: “It so surreal. Something you never thought would happen to your own child. You almost can’t bear it”. Providing information about services and self-care strategies for informal providers was therefore considered important. Exercising self-care was not easy, however: “Self-care is very difficult. It’s almost something you need to be forced to do. So that could be something to focus on [on website]” (Helen). Linda similarly explained that she did not spend much time focusing on her own reactions and needs, because her daughter’s needs had been her primary focus: “You think so much about them [child] in all of this. So, you don’t really know how you feel yourself”.

Both informal support providers encountered difficult choices and dilemmas concerning how to best parent and support their children following the assault. For example, each mother struggled to find a balance between respecting her child’s autonomy (e.g., desire not to engage with authorities) and having to make difficult decisions about what she believed was right for her child as their parent. According to Helen:

What the hell should I do? Because she’s not well. She has all these things with restricting food and cutting herself. I almost can’t stand having to wait until the house of cards tumbles down. I wish I could kick it down.

In this context, both informal support providers experienced feelings of self-doubt and self-blame, as well as judgment from others for the assault of their child and its aftermath. According to Helen: “What you have to face from the outside world is also extremely tough”. Addressing these difficult choices and feelings among informal providers on the website was therefore recommended.

### Theme 4: labels matter

The website translates to “rape.dk” and various terms were used on the website to refer to sexual assault (e.g., “rape”) and those who have been sexually assaulted (e.g., “victim”). While formal support providers were uncertain about what terms they preferred in this context, survivors and informal support providers reacted strongly to this topic. According to Julie (survivor): “It was difficult to search for those words. I didn’t want to touch them. I didn’t want to look at “rape” and “assault”. I didn’t want them in my search history”. In the beginning, it was therefore “difficult to be on a website called [rape.dk]”. Helen (mother) similarly explained that: “It’s such a vile word [rape]. I can’t get it over my lips. I mean, I still can’t [1 year later] (…) It could even prevent me from contacting [the SAC]. Because it’s called ´ Something-Rape-Victims ´ ”. Finding better alternatives was not easy, however: “I don’t know what else to call it (…) It is also good that it is concrete” (Julie)*.* Both survivors preferred “people affected by sexual violence” to “victim” or “survivor”, because the sexual assault was something that happened, not their identity. Formal support providers furthermore noted that the website was generally geared towards survivors identifying as women due to frequent use of she/her in relation to survivors and wondered if the website sufficiently embraced other gender identities. When other genders were mentioned, it was done using the binary “he/she”.

### Theme 5: requested features

Participants of all types requested more interactive elements on the website such as podcasts, videos, animations, and chat. Desired topics for audio and video material included information about rape myths and common trauma reactions, self-help-strategies, and how to access different kinds of formal support. Several participants believed that having more interactive elements would increase both the utility and reach of the website. For example, formal support providers requested videos/animations covering essential elements of treatment that could be used in or between sessions (e.g., exposure therapy and breathing exercises).

Survivors appreciated the testimonies of other survivors and wanted more content from survivors’ point of view: “Reading academic texts about [sexual assault] is one thing, but reading about [sexual assault] from someone who has experienced it themselves and being able to relate is something else” (Louise). However, both survivors pointed out that current testimonies lacked diversity in terms of the characterization of survivors. In this context, Julie (survivor) added that real accounts were even more powerful than fictional ones. Julie felt that having survivors share their real identify (e.g., name, face) would be most effective, especially if it was possible to interact with the people “behind the story” through the website. Alternatively, Julie proposed creating (online) forums and communities for survivors to help survivors connect: “I kind of had a need to talk to others who had experienced it [assault] (…) I also had a bigger need to talk about it than my friends and family could accommodate”. Depending on how it was set up, Julie did see disadvantages to online forum, however: “I would probably be afraid if anyone could log in, because someone could have bad intentions” and “You never know who is on the other side”. Lastly, a survivor (Julie) questioned whether websites are still contemporary and therefore advised the SAC to have a social media presence: “That’s a nice and easy media and what many people use today instead of typing in a website address”.

## Discussion

The present study sought to identify the experiences and needs of different website users (survivors and informal and formal support providers). Interviews were analyzed using thematic analysis, resulting in five themes. In the following section, themes are discussed and implications for practice and research are provided. We then provide a brief description of the new SAC website, reflect on the use of action research, and highlight some limitations of the present study.

In the first theme (*Website Function*), we found that survivors and support providers use the internet to locate information about sexual assault and available supports. This is not surprising, as many other studies have emphasized that survivors often find eHealth more accessible and less intimidating than reaching out to physical services directly (e.g., Carretta et al., 2016). More interestingly, however, we found that survivors used the SAC website to define their experience as sexual assault and to evaluate whether their distress was caused by sexual assault. While online health information seeking and self-diagnosis is a growing phenomenon (Farnood et al., 2020), little research has specifically investigated survivors’ use of the internet to determine whether an experience “counts” as sexual assault and how survivors “should” react to such acts. Additionally, survivors in the present study found information on the website to be more credible than that from their personal network, underscoring the relative power such websites have in shaping perceptions of sexual assault. Much care is therefore needed when describing sexual violence and its aftermath to avoid reinforcing misconceptions and stereotypes. Relatedly, websites and other eHealth platforms must keep up with evolving views on sexual violence, which require continuous updating. For example, the implementation of consent-based rape laws in Denmark in 2021 changed both legal and societal perceptions of sexual assault, requiring extensive changes to the website and public education. Denmark has previously faced significant criticism from multiple parties for the barriers survivors encounter when seeking justice - including the prior legislative emphasis on violence and coercion (e.g., Amnesty International, 2019). In contrast, the new law centers on sexual autonomy and consent, marking a pivotal shift in how sexual violence is conceived. This reform has largely been contributed to activism from survivors and organizations including Amnesty International, and has improved, but not eradicated, barriers to justice for survivors (Uhnoo et al., 2024).

In the second theme (*Helpful Content*), we found that participants viewed sexual assault websites as helpful. This is in line with the findings of a recent systematic review on eHealth for sexual assault, which concluded that different forms of eHealth, including websites, are generally perceived as beneficial by users (Bach et al., 2023). Faulty perceptions about sexual assault persist in society and feelings of shame and blame continue to plague survivors in the present study and beyond (Bhuptani & Messman-Moore, 2019; Kennedy & Prock, 2018). Information to counter rape myths was therefore seen as particularly helpful by participants of all kinds, highlighting the educational role of the website. This is likely an important component of most, if not all, eHealth applications for sexual assault, as research suggests that rape myth acceptance is still relatively high internationally and in Denmark, especially among young males (Det kriminalpræventive råd, 2023). General advice and self-help strategies for survivors were also considered helpful but could also make survivors feel inadequate and pressured (e.g., if phrased as a checklist). Well-intentioned information and advice can therefore have the opposite effect if not carefully worded.

Theme 3 (*Unhelpful Content*) reported that participants found the website difficult to navigate and comprehend. Several studies indicate that the reading level required to read most sexual assault websites in the United States is equivalent to the reading level required to read *Time* or *Newsweek* (Sorenson et al., 2014; Taylor, 2018). While this is problematic for a large segment of people, it can be particularly problematic for sexually victimized persons who may be severely distressed (Hayes et al., 2012). Marginalized groups of all kinds, including language minorities and people with disabilities, face higher risks of sexual assault, further highlighting the need for simple, accessible websites (Ullman & Najdowski, 2011). Despite this, most research on eHealth for sexual assault has not evaluated or addressed accessibility for marginalized survivors, who appear to be underserved not only by traditional services but also by eHealth services (Bach et al., 2021; Bach et al., 2023). Therefore, much care is needed in terms of wording and structure of eHealth applications for sexual assault, including decisions regarding fonts, text size, color contrasts, visual aids, and other features that impact readability and comprehension.

Another significant point in Theme 3 is that the original website did not adequately include the role and needs of informal support providers, including parents of (young) sexual assault survivors. This is unfortunate because parents and other informal support providers can be instrumental in furthering or hindering survivors’ healing process (Ahrens, 2006). Informal support providers, including parents, can also experience significant distress, as evident in this study. Furthermore, parents face difficult choices regarding how to best support their child following a sexual assault, especially when parents and children have different views on what actions should be taken after the assault (e.g., whether to involve authorities or not). eHealth resources should therefore not only provide information and support to survivors but also their loved ones. Very little, if any, research has looked at informal providers’ experiences and needs when using eHealth for sexual assault. More research in this area is therefore warranted.

Theme 4 (*Labels Matter*) focused on the impact of labels, naming, and language use on the website. While the literature on trauma informed care and language is extensive (e.g., Reeves, 2015), very little has been written, on the naming of sexual assault services (and websites). While terms like “sexual assault” and “rape” might be viewed by some service users as stigmatizing and triggering, others may find them factual and concrete. Internationally, the names of organizations (and websites) appear to vary. While some do not include explicit reference to sexual violence others do. Many titles are also abbreviations, including well-known US organization and website “RAINN” (“Rape, Abuse & Incest National Network”). Of note, survivors in this study did not identify with the terms “victim” and “survivor”. Recent research similarly suggests that labels like “victim” and “survivor” play a central role in shaping attitudes toward sexual assault, and that there is a need to further explore and potentially rethink sexual violence labels (O’Shea et al., 2024). Theme 4 also indicated that the original SAC website catered mostly to women and used binary language (he/she). While women are at a higher risk of sexual assault, omitting males and using binary language in relation to gender is problematic because it can reinforce stereotypical views about sexual assault and exclude certain survivors. This is likely an important issue on many sexual assault websites, as most eHealth applications for sexual assault appear to focus on women and not all include male and LGBT + perspectives (Bach et al., 2023). Attention to diversity is therefore needed when developing eHealth for sexual assault.

Theme 5 (*Requested Features*) demonstrated a desire for interactive elements on the website. While videos can be administered and distributed to a large segment at low cost, the production of videos and other interactive materials can be both time-consuming and costly as creative and technical specialists are often needed. There is relatively little research on the effect of (intervention) videos for survivors, but there is some evidence suggesting that videos can be effective in reducing assault-related distress and, for example, substance misuse (e.g., Gilmore et al., 2019; Resnick et al., 2012; Walsh et al., 2017; Walsh et al., 2021). Theme 5 also reported a desire to incorporate chat services on the website. As with videos, developing and maintaining secure chat can be costly, as previous research has demonstrated security related challenges (Schrag et al., 2021) and that around the clock monitoring is often necessary to protect users from harassment and abuse (Webber & Moors, 2015).

**Figure 1.**
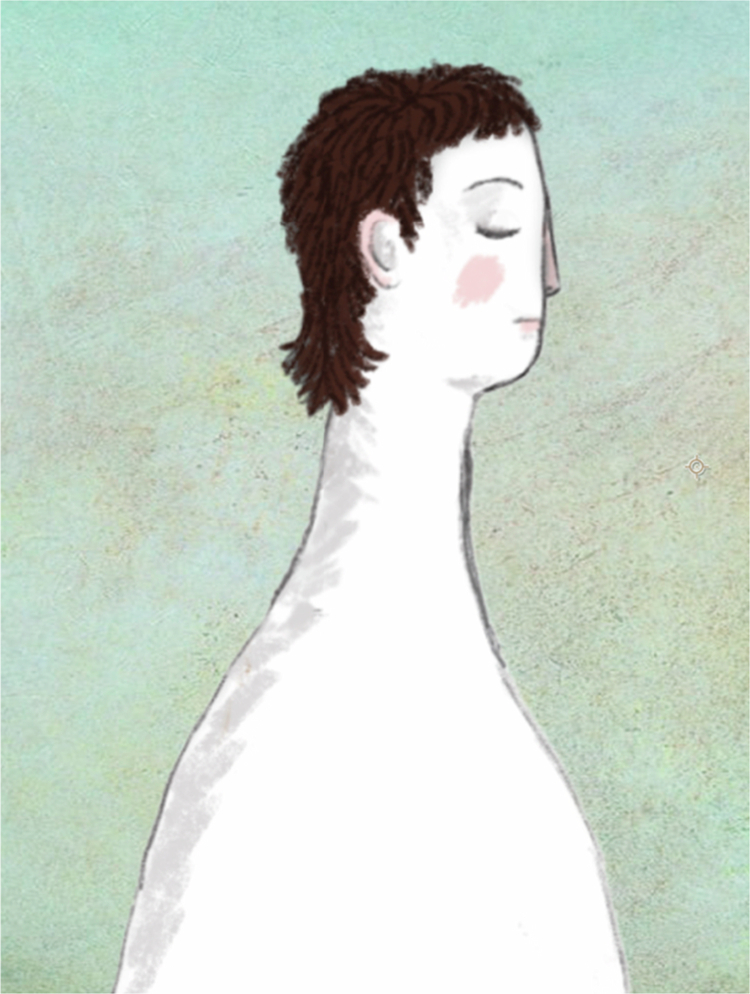
Example of digital art on website. Copyright: The authors.

The present study could have resulted in the abandonment of the SAC website in favor of other eHealth applications, but instead the study underscored the continued need for a national sexual assault website. Taken together, the results emphasized a need for a more contemporary, accessible, and inclusive website. To this end, we developed a new, mobile friendly website and incorporated several accessibility enhancing features (e.g., adequate color contrast ratio between text and background, texting of videos, readable fonts, changeable font sizes, read-aloud function, translation options). We also selected a more minimalistic design scheme that was easier to navigate and appeared more modern. Text was reduced as much as possible, and we utilized additional modalities including animations and live action videos. The live action videos featured various professionals in the multidisciplinary team (psychologists, police, lawyers, forensic nurses and doctors) and involved meetings and interviews with new participants, demonstrating the dynamic nature of action research. It was also important to convey in text and images that sexual assault is perpetrated across all intersections of identity, and we therefore utilized a broad definition of sexual violence, explained that sexual assault can happen to anyone, and emphasized that sexual assault is never the survivor’s fault. In addition, animations of people were designed to be abstract enough to make their gender, race, body type, and so forth less obvious, making them relatable to a broader audience, while simultaneously maintaining enough naturalism to clearly convey emotive expression (see Figure 1 for example). Although primarily in Danish, the website and videos can be freely accessed at www.voldtaegt.dk for a more detailed view of all its contents and features.

Action research requires a high degree of collaboration and joint decision-making, which was both highly awarding as well as challenging and time-consuming. For example, different team members have different priorities that can be difficult to reconcile (e.g., how to prioritize “traditional” research outcomes such as scientific publications versus concrete “products” such as the website). Nevertheless, we believe that the action research process was invaluable. Most importantly, website users could provide first-hand knowledge on the topic. Researchers and SAC employees, in turn, provided research- and practice-based knowledge on eHealth for sexual assault. While researchers could organize research activities and manage the overall project, the SAC employee could navigate the local context (a large university hospital) and make decisions on behalf of the SAC, which was essential in securing the implementation and maintenance of the new website. While we are grateful that the hospital permitted and supported the current study, the research was shaped by the demands and practices of the hospital (e.g., needing hospital approval to omit medicalized language on the website, such as not wanting to address survivors as “patients”). Other researchers have noted similar challenges in relation to conducting various kinds of qualitative research in hospital settings, especially on sensitive topics, such as sexual assault (Parnis et al., 2005).

This research is not without limitations. Although survivors corresponding to the demographic at highest risk of sexual assault (young women) were recruited, we were not successful in recruiting survivors and other participants of more diverse genders. In addition, informal providers consisted only of mothers and formal providers mostly of psychologists. Finally, recruitment was conducted at a SAC, limiting the perspectives of those who find SACs inaccessible or have chosen not to seek formal support. This lack of diversity limits the breadth of generated user needs, and thereby also the utility of the presented user needs as well as the end product (the website). The website was, however, informed by multiple sources.

To summarize and conclude, the present study used action research to co-develop a national sexual assault website in Denmark with survivors and support providers. Results indicate that websites are used to define sexual assault and its aftermath (Theme 1), that information to counter rape myths is essential (Theme 2), that websites must include all those affected by sexual violence, not just survivors (Theme 3), that terminology and labels matter (Theme 4), and that interactive materials are desired (Theme 5). The website has been revised according to these findings and insights from the extant literature, resulting in a more contemporary, accessible, and inclusive website. The present study also demonstrated that action research in this area is time-consuming but feasible, and that survivors and other stakeholders can contribute to the development of new interventions for sexual assault. Future research on this topic would benefit from using more diverse populations as well as other types of support providers (e.g., romantic partners). Nevertheless, we hope that the present paper will inspire others seeking to develop eHealth for sexual assault or those wanting to conduct collaborative research in this area, which is highly recommended.

## Ethical statement

All necessary ethical and legal approvals according to Danish legislation were granted for conducting the present study. Written informed consent to participate in the study and written consent to publish interview quotes have been obtained from all participants.

## Data Availability

Data to support the study results are provided in the manuscript (direct interview quotes). Due to the sensitivity of the material, complete data sets are not openly available (e.g., full transcripts, audio recordings).
